# Environmental and Management Effects on Demographic Processes in the U.S. Threatened *Platanthera leucophaea* (Nutt.) Lindl. (Orchidaceae)

**DOI:** 10.3390/plants10071308

**Published:** 2021-06-28

**Authors:** Timothy J. Bell, Marlin L. Bowles, Lawrence W. Zettler, Catherine A. Pollack, James E. Ibberson

**Affiliations:** 1 Department of Biological Sciences, Chicago State University, 9501 S King Dr., Chicago, IL 60628, USA; tbell22@csu.edu; 2 The Morton Arboretum, 4100 IL 53, Lisle, IL 60532, USA; mbowles@mortonarb.org; 3 Department of Biology, Illinois College, 1101 W College Ave, Jacksonville, IL 62650, USA; ibberson.james@ic.edu; 4 U.S. Fish and Wildlife Service, 230 South Dearborn St., Suite 2938, Chicago, IL 60604, USA; cathy_pollack@fws.gov

**Keywords:** climatic variability, demographic modeling, fecundity, fire management, habitat, pollination, crossing, survivorship, viability

## Abstract

Populations of the U.S. threatened orchid, *Platanthera leucophaea*, are restricted to fragmented grassland and wetland habitats. We address the long-term (1998–2020) interactive effects of habitat (upland prairie vs. wetland), fire management (burned vs. unburned) and climatic variation, as well as pollination crossing effects, on population demography in 42 populations. Our analysis revealed the consistent interactive effects of habitat, dormant season burning, and climatic variation on flowering, reproduction, and survival. Burning increased flowering and population size under normal or greater than normal precipitation but may have a negative effect during drought years apparently if soil moisture stress reduces flowering and increases mortality. Trends in the number of flowering plants in populations also correspond to precipitation cycles. As with flowering and fecundity, survival is significantly affected by the interactive effects of habitat, fire, and climate. This study supports previous studies finding that *P*. *leucophaea* relies on a facultative outcrossing breeding system. Demographic modeling indicated that fire, normal precipitation, and outcrossing yielded greater population growth, and that greater fire frequency increased population persistence. It also revealed an ecologically driven demographic switch, with wetlands more dependent upon survivorship than fecundity, and uplands more dependent on fecundity than survivorship. Our results facilitate an understanding of environmental and management effects on the population demography of *P*. *leucophaea* in the prairie region of its distribution. Parallel studies are needed in the other habitats such as wetlands, especially in the eastern part of the range of the species, to provide a more complete picture.

## 1. Introduction

The orchid family (Orchidaceae) is one of the world’s largest plant families, with species represented in tropical, temperate, and boreal regions. Because of their complex requirements for pollinators and mycorrhizal fungi, orchids are often habitat-specific and adapted to narrow environmental and climatic conditions [1]. Many of these species are vulnerable to increasing direct human impacts, climate change, and altered fire regimes, though relatively few have been assessed for listing status, protection, and conservation needs [2,3].

To conserve the orchid species of north temperate plant communities, European and North American researchers have sought to understand their ecological adaptations, demographic responses to environmental dynamics, and vulnerability to human impacts. For example, in Canada, co-occurring *Platanthera blephariglottis* (Willd.) Lindl. and *Platanthera clavellata* (Michx.) Luer were found to partition habitats by different water levels, but not soil chemistry [4]. In Poland, *Platanthera bifolia* (L.) Rich. population demographics differed between natural and formerly human-occupied habitats, with greater reproduction under higher light regimes [5]. In Azerbaijan, *Platanthera chlorantha* (Cust.) Reichenb. had wide ecological variability and population structure among natural and disturbed habitats, and preferred a temperate, warm, and humid climate under specific soils [6]. Climatic variation may drive demographic processes. A late frost event caused demographic decline of *Ophrys argolica* subsp. *biscutella* (O. Danesch and E. Danesch) Kreutz in South Italy [7]. Precipitation in the previous and current flowering years corresponded to flowering and reproduction of *Platanthera hookeri* (Torr.) Lindl. in Canada [8], as well as *Dactylorhizal traunsteineri* (Saut. ex Rchb.) Soo in northern Europe [9]. In the U.S. and Canada, precipitation thresholds during different phenological stages affect the flowering and reproduction processes of *Platanthera praeclara* Sheviak and Bowles [10,11] and the closely related *Platanthera leucophaea* [12]. As these species occupy fire-dependent grassland vegetation, additional monitoring is needed to assess fire impacts and interactions with precipitation [13]. Grazing by domestic and wild ungulates may negatively impact these and other showy *Platanthera* species (e.g., [14]) as well as *Cypripedium calceolus* L. in Italy [15]; however, grazing reduced forb competition with *Dactylorhiza viridis* (L.) R.M. Bateman, Pridgeon, and M.W. Chase in the UK [16].

Collectively, these studies support the contention that long-term monitoring data linked with drivers of plant population dynamics are needed to improve our understanding of plant demography and provide accurate forecasting of extinction risks [17]. This need is critically important for endangered plant species that persist in fragmented habitats, such as grasslands in central North America. Orchid species occupying these habitats are particularly vulnerable to habitat loss and degradation from grazing and fire suppression, as well as disruption of pollination processes, inbreeding depression, and adverse impacts on associated mycorrhizal fungi [18,19,20,21,22,23]. Few studies include the robust long-term monitoring data required to understand ecological and genetic factors driving demographic processes for such plants [24,25,26,27].

**Figure 1:**
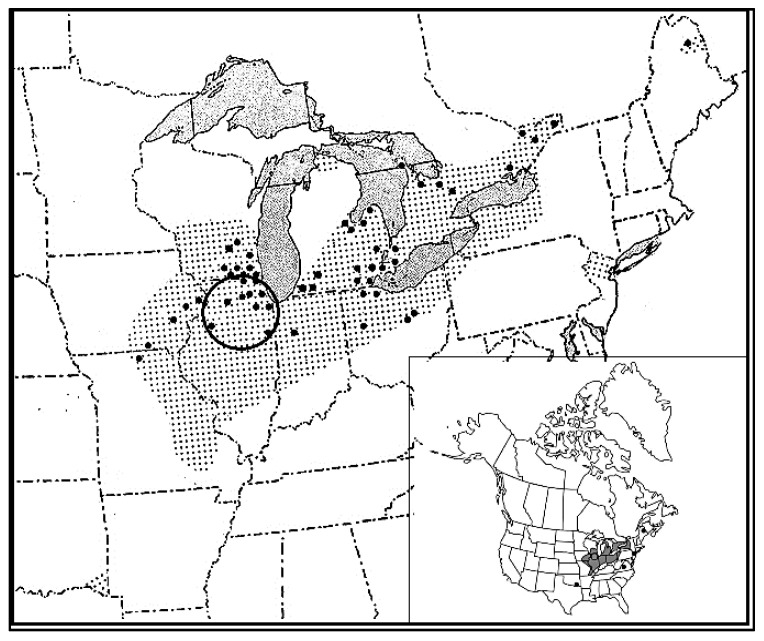
Distribution of *Platanthera leucophaea* in Midwest and Eastern North America. Dot grid represents former distribution, large dots represent U.S. counties and Canada municipalities with one or more extant populations. Ellipse indicates study area. Inset: distribution in North America. Reproduced from www.eFloras.org (19 March 2021) with permission.

In this paper, we address long-term interactive effects of habitat (upland prairie vs. wetland), fire management (burned vs. unburned), and climatic variation, as well as pollination crossing effects, on population demography of *P. leucophaea* in 42 populations in fragmented grasslands and wetlands of the Chicago region of northeastern Illinois in the United States (Figure 1). Based on anecdotal information, we expect a positive effect of fire on flowering and fecundity [28]. However, prairie and wetland populations may respond differently to fire and climatic variation if greater access to ground water in wetlands buffers against drought stress encountered in upland prairies [29,30]. This effect may be more apparent if greater soil moisture loss occurs following fire in upland sites than in wetlands. Moreover, *P. leucophaea* is also vulnerable to inbreeding in small populations (see below), which could affect population growth and persistence in fragmented habitats. Specifically, we ask the following questions:

(a) How do long-term trends in sizes of flowering plant populations correspond to habitat and climatic variation?
(b) How are flowering and fecundity affected by habitat and fire, and how do they interact with climatic variation?
(c) How is plant survival affected by habitat and fire, and how do these affects interact with climatic variation?
(d) How do crossing rates (selfing and outcrossing within and among populations) affect germination?
(e) What are the effects of these factors on population demography?

### 1.1. Study Species

The eastern prairie fringed orchid, *Platanthera leucophaea* (Nutt.) Lindl. is native to midwestern and eastern North America where it is listed as threatened in the United States [31] and endangered in Canada [32]. The International Union for Conservation of Nature Red List of Threatened Species ranks *P. leucophaea* as a species of least concern [33]. This species’ former distribution extended from the Mississippi valley eastward through the lower Great Lakes states and adjacent Canada, with outliers to the east and southwest (Figure 1). Its primary habitats are tallgrass prairie and sedge meadow in the western part of its range, and open sedge meadows, fens, sphagnum bogs and similar wetland habitats eastward [18,28,34]. In the U.S., it persists in 96 populations in eight states [35] and in Canada, about 16 populations are known in southern Ontario [32]. Most populations have been reduced in size by habitat fragmentation and by the deterioration of vegetation caused by fire exclusion and disrupted hydrology. Some populations remain vulnerable to herbivory by eastern white tail deer as well as poaching of flowering plants.

**Figure 2:**
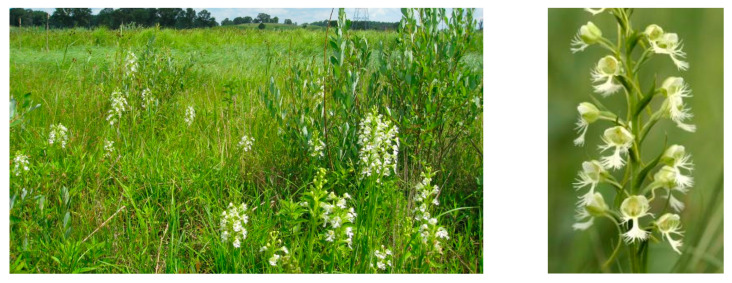
**Left**—Sedge meadow habitat of *Platanthera leucophaea* in northern Illinois. Photo: U.S. Fish and Wildlife Service. **Right**—*P. leucophaea* inflorescence illustrating fringed labellum and long nectar spurs. Photo: D. Kurz.

**Figure 3:**
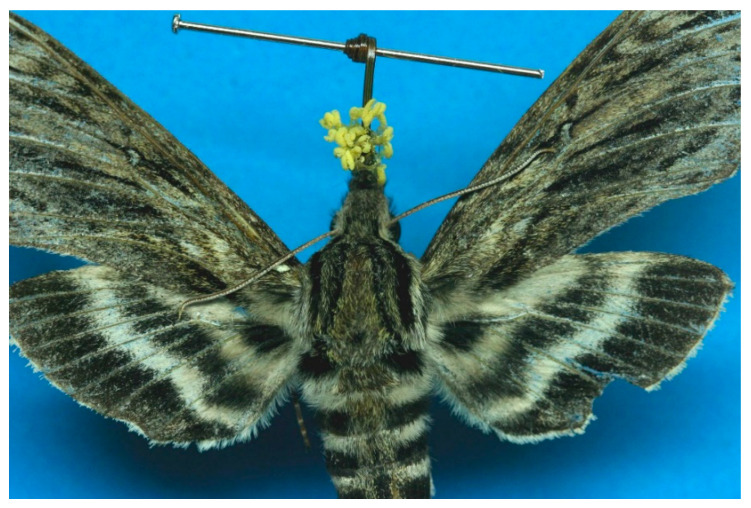
The hawk moth, *Lintneria eremitus* Hübner with hemipollinaria of *Platanthera leucophaea* affixed to the base of the insect’s proboscis. Specimen from Jackson Co., Iowa. Photo: R. Panzer.

This terrestrial perennial herb flowers in early to mid-summer. It produces a showy solitary inflorescence of up to 100 (mean = 12.4 + 0.07 se) white, nocturnally fragrant flowers adapted to hawk moth (Sphingidae) pollination [18,28,36,37] (Figure 2). The labellum is fringed, 18 mm (0.43 se) long and 20.4 mm (0.54 se) broad; the nectar spur is 35.6 mm (0.89 se); pollinaria (hemipollinaria *sensu* Dressler [38]) are positioned parallel on either side of the nectar spur entrance, with viscidia facing and separated by 1.2–3.2 mm to allow deposition on the proboscis of pollinators [28]. Five species of hawk moths have been identified as pollinators of *P. leucophaea*: *Lintneria eremitus* Hübner [37,39,40] (Figure 3), *Eumorpha achemon* Drury [39,41], *E. pandorus* Hübner [39], *Xylophane tersa* Linnaeus [42], and *Manduca sexta* Linnaeus [28]. This species has a facultative outcrossing breeding system and low genetic differentiation among populations due to hawk moth pollination, and it is vulnerable to inbreeding depression in small populations [20,36,43].

**Figure 4:**
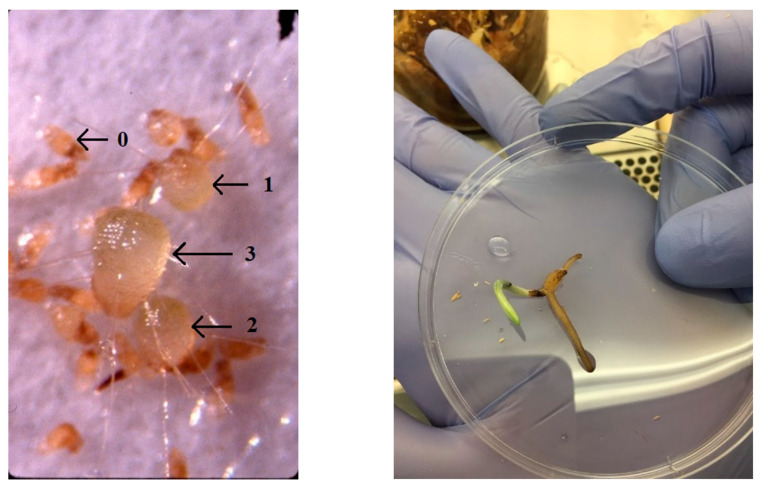
**Left**—Early growth stages of leafless mycotrophic seedlings (protocorms) cultured with mycorrhizal fungi in vitro (see methods). The larger protocorm in the center of the image is now at Stage 3 and measures only 3 mm long. **Right**—*Platanthera leucophaea* Stage 5 seedling initiating a green strap leaf following in vitro germination using *Ceratobasidium*. Cold treatments were applied prior to germination, and after shoot initiation in darkness during 1st year. Photo: E. Esselman.

Fruit (capsule) maturation and seed set occur in early September. Seeds are dark brown, monoembryonic, dust-like, and wind-dispersed. Seed germination is facilitated by a cold-moist stratification lasting at least two months [44]. Seedling development to the protocorm stage is subterranean (ca. 5–15 cm depth) and facilitated by contact with *Ceratobasidium* (Figure 4) [44]. Protocorms (Figure 4) remain underground for 1–3 years as obligate mycotrophs until they initiate a solitary strap leaf early in the growing season (Figure 4). The mature root system consists of a starch-filled elongated tuber positioned beneath 3–10 narrow, brittle lateral roots that harbor mycorrhizal fungi, mostly free-living saprophytes in the genus *Ceratobasidium* (Basidiomycota, Cantharellales) [45,46,47]. The persistence of *Ceratobasidium* in roots, even after leaves are formed, suggests that *P. leucophaea* augments photosynthesis with mycotrophy throughout its life.

### 1.2. Study Area

This study took place primarily in the Chicago region of northeastern Illinois, U.S. The climate is continental, with average temperatures ranging from −6.05 °C in January to 22.33 °C in July, and 84.9 cm annual precipitation [48]. Prior to European settlement, lightning and fires set by Native Americans maintained tallgrass prairie in a region in which annual rainfall can support forest vegetation [49]. The area was deglaciated about 12,000 years BP, leaving glacial moraines and outwash features interspersed with glacial lakes and the former bed of glacial Lake Chicago [50]. Upland soils are fine-textured clay and sandy loams, while wetlands occur on peat and muck soils [51]. Most of the naturally occurring prairie and wetland remnants are 0.5–50 ha in size and isolated in a former agricultural matrix subjected to ongoing urban sprawl from the Chicago region’s population of ~9.5 million people. Prairie habitats have deteriorated from fire suppression which allows succession to woody vegetation and loss of herbaceous plant diversity. Many of these areas are now managed with dormant season prescribed fire [52]. Wetland habitats are threatened by invasive plant species, altered groundwater discharge, and eutrophication as well as fire exclusion [52]. Eutrophication can also negatively impact orchid mycorrhizal relationships [53].

## 2. Results

### 2.1. Long-Term Trends in Prairie and Wetland Populations

**Figure 5:**
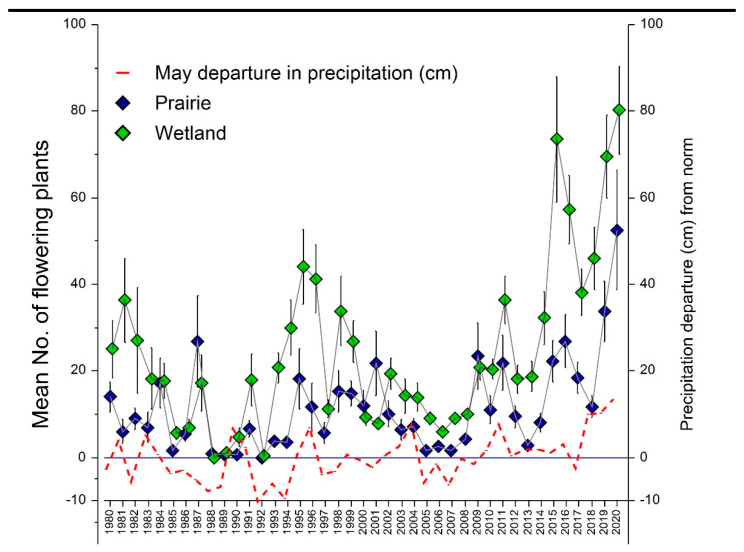
Chicago region *Platanthera leucophaea* population trends in prairie and wetland habitats, and departure of May precipitation from the 1980–2020 norm. Means represent annual flowering plant census ± se × 0.50. Replications: Prairie N = 16, Wetland N = 22. Correlations: prairie vs. wetland (Pearson’s R = 0.7621, t = 7.35, *p* < 0.001), May precipitation vs prairie (Pearson’s R = 0.5062, *t* = 3.67, *p* = 0.001), May precipitation vs wetland (Pearson’s R = 0.5831, *t* =4.48, *p* < 0.001), prairie response to previous September precipitation (Pearson’s R = 0.4070, *t* = 2.75, *p* = 0.009), wetland response to previous September precipitation (Pearson’s R = 0.1090, *t* = 0.68, *p* = 0.503). Population size ANOVA: prairie vs wetland habitat (F = 15.62, *p* < 0.001), precipitation departure above and below norm (F = 15.95, *p* < 0.001), habitat x departure (F = 4.42, *p* = 0.039).

Long-term trends in prairie and wetland flowering populations were cyclic and significantly correlated (Figure 5). Trends for both habitats were also correlated with May precipitation, and the trend in prairie habitat was significantly correlated with September precipitation during the previous year. Prairie habitats were also correlated with Palmer Drought Severity Index (PDSI) values for May (Spearman’s R = 0.3642, *p* = 0.0315, June (Spearman’s R = 0.3804, *p* = 0.0242), and July of the previous year (Spearman’s R = 0.474, *p* = 0.0046). Likewise, wetland habitats were correlated with PDSI values for May (Spearman’s R = 0.5008, *p* = 0.0022, June (Spearman’s R = 0.5815, *p* = 0.0002), and July of the previous year (Spearman’s R = 0.318, *p* = 0.0669). Wetland flowering populations were more than twice as large as prairie populations. Both prairie and wetland populations were larger when May precipitation exceeded the norm than when it was lower than the norm. However, wetlands had a twofold greater response to increased precipitation. Wetlands increased 134% from 14.4 (2.56 se) to 33.7 (4.67 se) flowering plants when the May departure exceeded the norm, while prairies increased only 70% from 8.6 (1.90 se) to 14.6 (2.61 se) flowering plants.

### 2.2. Fire, Habitat and Climatic Effects on Flowering and Fecundity

**Figure 6:**
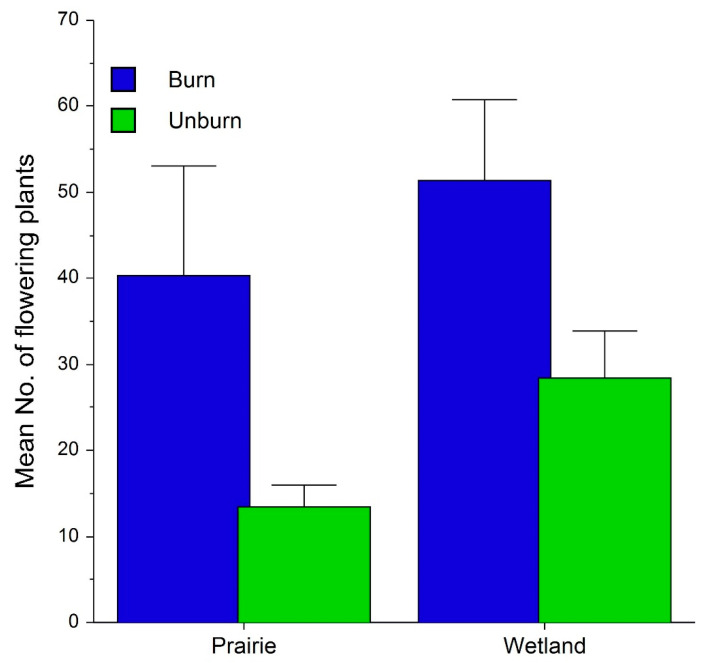
Fire and habitat effects on mean (+se) annual *Platanthera leucophaea* population sizes. Means represent annual flowering plant census numbers. ANCOVA: May precipitation (F = 11.65, *p* < 0.001), previous September (F = 6.41, *p* = 0.012), previous July PDSI (F = 3.36, *p* = 0.068), previous August PDSI (F = 3.41, *p* = 0.066), habitat (F = 28.73, *p* < 0.0001), fire (F = 3.68, *p* = 0.056). There were no significant interactions.

**Figure 7:**
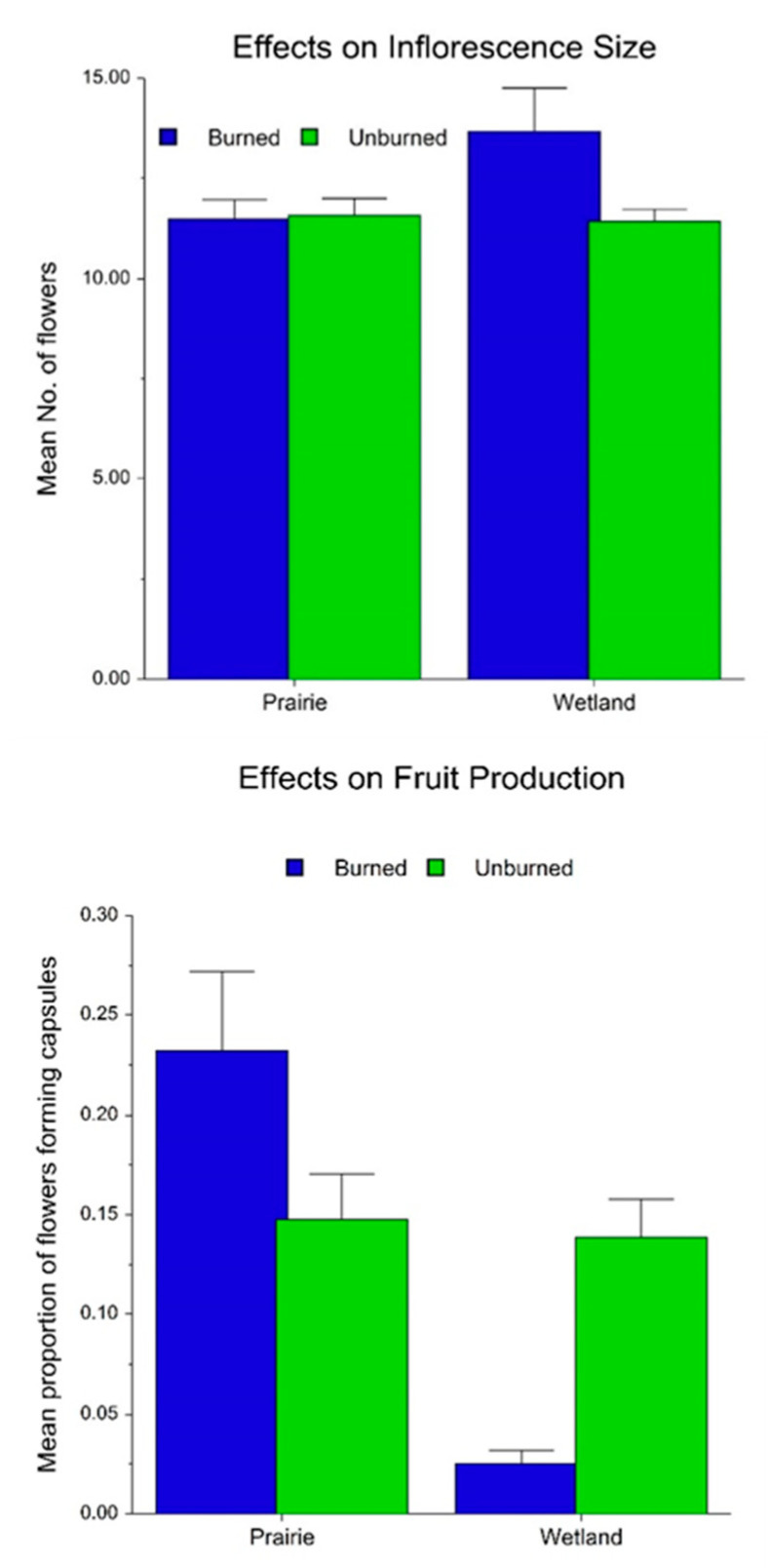
Fire and habitat effects on mean (+se) *Platanthera leucophaea* inflorescence sizes (left) and proportional seed capsule production (right). ANCOVA inflorescence size: growing season PDSI (F = 12.04, *p* < 0.001), habitat (F = 0.70, *p* = 0.404), fire (F = 9.03, *p* = 0.003). ANCOVA pod production: growing season PDSI (F = 13.76, *p* < 0.001), habitat (F = 5.87, *p* = 0.016), fire (F = 7.44, *p* = 0.0078), habitat x fire (F = 35.63, *p* < 0.001). Only significant interactions presented.

The average number of flowering plants was greater in wetland than in prairie habitats, and was greater in burned than unburned habitats (Figure 6). The current year’s May precipitation and September precipitation of the previous year were significant covariates with these results. July and August PDSI of the previous year were also marginally significant covariates. Inflorescences were also significantly larger in burned habitats, and growing season PDSI covaried with this effect (Figure 7). Although there was no significant interaction, this difference is more apparent in wetland habitat. The proportion of flowers converted to seed capsules by open pollination was significantly greater in prairie than in wetland habitat (Figure 7). As indicated by the significant interaction, this proportion was greater in burned than in unburned prairie habitats, but greater in unburned than in burned wetland habitats.

### 2.3. Fire, Habitat and Climatic Effects on Survival

**Figure 8:**
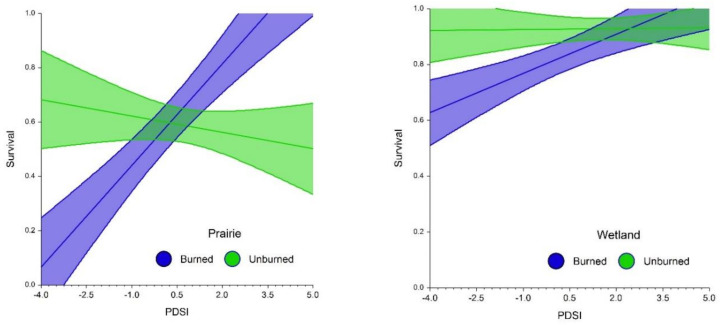
Climate, habitat, and fire effects on *Platanthera leucophaea* survival probability in prairie (left) and wetland (right). Bands represent 95% C.I. Logistic regression: model R² = 0.32118, fire (Z = 1.444, *p* = 0.149), habitat (Z = 7.225, *p* < 0.001), fire x habitat (Z = 2.067, *p* = 0.039). PDSI (Z = 2.006, *p* = 0.045), PDSI × fire (Z = −4.274, *p* < 0.001), habitat × PDSI (Z = −1.304 *p* = 0.192).

In a Logistic Regression model, habitat, fire, and climate had significant main or interactive effects on survival (Figure 8). Overall, survivorship was greater in wetlands than in prairies but did not differ between burned and unburned habitat. However, there were significant interactions between fire treatments and both habitat and PDSI. In burned prairies, survival increased from about 0.1 to 1 with increasing PDSI. In burned wetlands, survival increased from about 0.6 to 1.0 with increasing PDSI. However, there was no response in unburned habitats to change in PDSI. These changes occurred across a moderate range of PDSI values, and thus do not reflect severe drought or wetness conditions.

### 2.4. Crossing Effects on Germination

**Figure 9:**
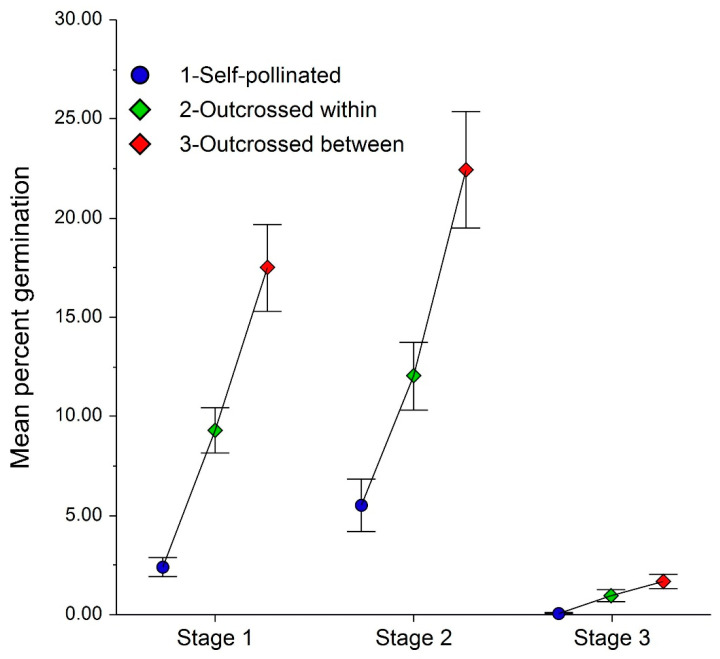
Crossing effects on mean (+se) *Platanthera leucophaea* seed germination. ANOVA Stage 1: year (F = 22.23, *p* < 0.001), Cross (F = 19.07, *p* < 0.001), year × cross (F = 5.16, *p* < 0.001. Stage 2: year (F = 42.24, *p* < 0.001), Cross (F = 8.53, *p* < 0.001), year × cross (F = 5.16, *p* < 0.001). Stage 3: year (F = 6.77, *p* < 0.001), Cross (F = 5.33, *p* = 0.005), year × cross (F = 2.56, *p* = 0.039).

*Platanthera leucophaea* seed germination to Stages 1, 2, and 3 differed significantly among crossing treatments, with lowest germination in self-pollinated plants and greatest germination in seeds resulting from outcrossing between populations (Figure 9). Percent germination also differed significantly among stages; it exceeded 15% in Stages 1 and 2 but was <5% in Stage 3. In Stage 4, germination was <0.005%, and it was <0.001% in Stage 5. In these stages, germination was too infrequent to allow statistical testing among crossing methods; however, outcrossing between populations tended to be higher, and self-pollination tended to result in lower germination percentages.

### 2.5. Effects on Population Demography

**Figure 10:**
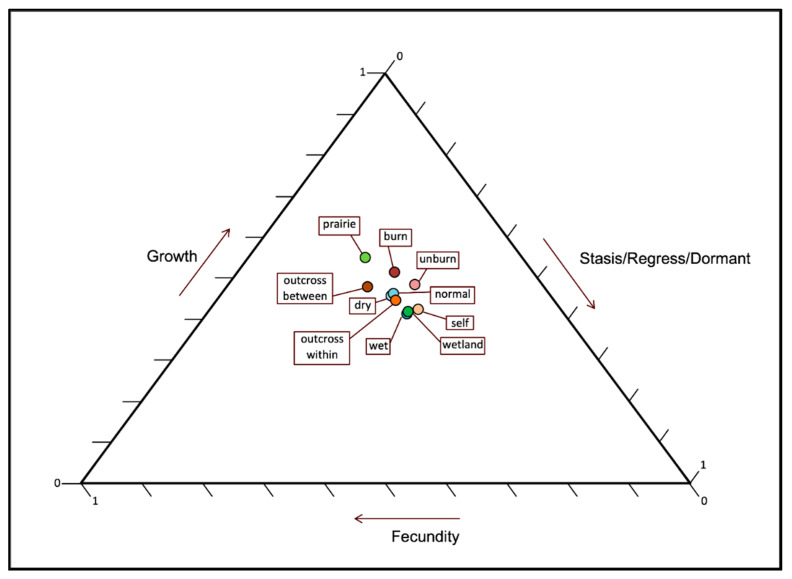
Ordination of *Platanthera leucophaea* growth, stasis/regression and fecundity elasticities for burn treatment, drought effects, crossing effects, and habitat type.

The pooled population growth rate (λ) for Illinois Eastern Prairie Fringed Orchid populations is λ _pooled_ = 1.11 (Table 1). Growth rates were significantly higher for populations in burned habitat (λ _burn_ = 1.63, λ _unburn_ = 1.11), with normal rainfall (λ _drought_ = 0.62, λ _normal_ = 1.11, λ _wet_ = 0.71), outcrossed between populations (λ _outcrossing btw pops_ = 1.73, λ _outcrossing within pops_ = 1.11, λ _selfing_ = 0.88) and wetland ((λ _wetland_ = 1.29, λ _prairie_ = 0.84) (Table 1). For the pooled transition matrix (indicated as “out win” in Figure 10), changes in growth have greater effects on λ compared to stasis or fecundity. The effects of changes in growth on λ were greater, and changes in stasis lower, in burned compared to unburned years. The effects of changes in stasis on λ were greater, and growth lower in wet years compared to dry or normal years. The effects of changes in fecundity and growth on λ increased with increasing outcrossing and stasis decreased with increasing outcrossing. Differences in the effects of changes in growth and stasis on λ were greatest between prairie and wetland habitats, with effects higher for growth and lower for stasis in prairies vs. wetlands. Demographic matrix analysis indicates that the mean age of parents of cohort juveniles is 3.98 years for the pooled demographic matrix.

**Figure 11:**
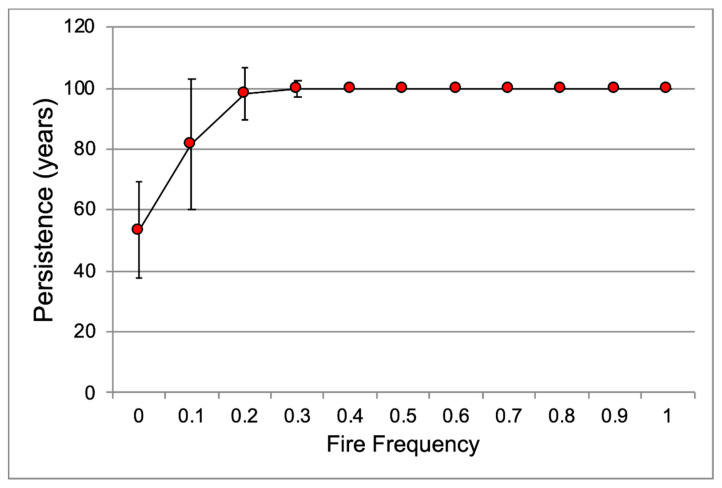
Population persistence (mean + sd) for *Platanthera leucophaea* by fire frequency (proportion of years burned).

The modeled Platanthera *leucophaea* population presentence increased with burn frequency (Figure 11). Burning at least 3 years out of 10 appears to be the minimum frequency to ensure population persistence for 100 years.

**Figure 12:**
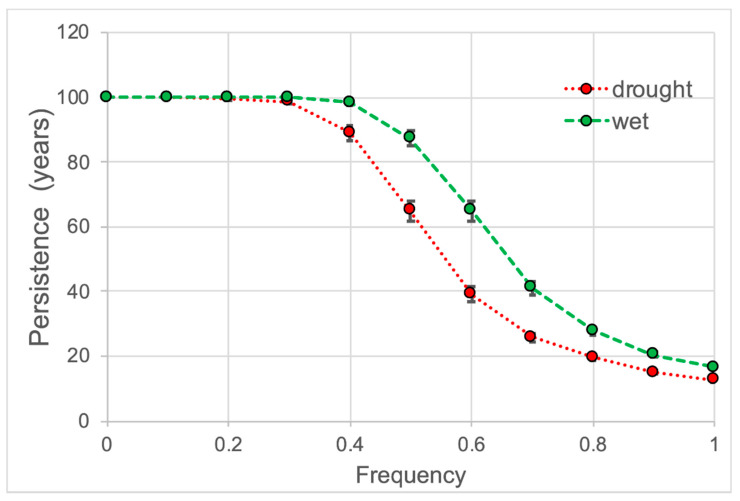
Population persistence (mean + se) for *Platanthera leucophaea* by drought and wet year frequency (proportion of drought or wet years).

Matrix modelling indicates that population persistence is high for years with normal PDSI (0.0 frequency) and begins to decrease with drought year frequency of 0.4 and wet year frequency of 0.5 (Figure 12). For drought and wet year frequencies of 0.9 and 1.0, populations are projected to persist for 20 years or less.

## 3. Discussion

According to Swarts and Dixon [2], terrestrial orchid conservation hinges on three actions: (1) the design and management of natural reserves, taking into account the specialized needs of the orchid; (2) the establishment of ex situ seed and fungus banks for orchids under immediate threat; and (3) the development of techniques for successful restoration. This study provides critical information for meeting the first action by strengthening our understanding of how demographic processes in *Platanthera leucophaea* respond to habitat and genetic management as well as climatic variation. Our data represent a long-term study replicated at the landscape level in fire-dependent vegetation at the western portion of the range of the study species. The findings may be less applicable to more eastern populations that occupy habitats where hydrology and succession may be more important drivers of population and vegetation dynamics. Nevertheless, the results of this study appear to be highly applicable to our study area. Moreover, the modeling applied compensates for gaps in direct fire frequency analysis and the data clearly show significant trends.

Our analyses reveal consistent interactive effects of habitat, dormant season burning, and climatic variation on flowering, reproduction, and survival of *Platanthera leucophaea*. This work reinforces other North American [8,10,11] and European [7,9] studies that have found multi-year climatic effects on demographic processes in orchids. Our work also reinforces the positive effects of burning on maintaining tallgrass prairie. By removing litter from the previous growing season, fire alters microclimate, increases nutrient availability to plants and soil microbes, and enhances plant physiological processes such as flowering [54]. As a result, most prairie plant species display positive responses to fire. Until now, empirical evidence that fire promotes flowering in *P. leucophaea* has been lacking. Following Pleasants [13], we hypothesized that many of the interactions found in this study are driven by effects on available soil moisture. Because burning removes litter, it allows soil moisture loss and increases water stress in plants if not compensated for by precipitation [29,30]. Thus, climate may regulate the effects of burning in prairies through rates of precipitation. This process also likely involves interactions with mycorrhizal fungi, which are more active following spring burns [55]. Thus, we suggest that fire effects are positive under normal or greater than normal levels of precipitation but may be negative during drought years if low available soil moisture induces stress leading to reduced flowering or mortality. The greater availability of soil moisture in wetlands would buffer this habitat from soil moisture stress, except during severe drought, and reduce interactions with fire. However, the variation measured by PDSI values for our data did not extend beyond moderate conditions, and more extreme wetland dynamics are driven primarily by water table fluctuations [56].

### 3.1. Long-Term Trends in Prairie and Wetland Populations

Our data indicate that long-term trends in the number of flowering plants in *Platanthera leucophaea* populations correspond to precipitation cycles. Morrison et al. [11] suggested that a precipitation threshold during emergence and growth is critical for flowering of *P. praeclara*. May precipitation appears to be most critical for driving this process in *P. leucophaea*, as years in which May precipitation falls below normal levels correspond to lower flowering population levels. September precipitation of the previous year also appears important and may correspond to the development of flowering primordia for the next growing season [18]. The positive correlations of PDSI with population fluctuations support this argument as well, though their lack of precision incorporates a greater monthly range. The lack of a significant September correlation in wetland habitat may be a response to greater access to groundwater in wetlands, and less dependence upon late summer precipitation for groundwater recharge. In wetlands, the more dynamic response of flowering plant numbers to precipitation cycles that exceeded the norm is probably due to a greater number of plants that survive drought in a non-flowering or dormant state [12]. Though wetland populations appear to have greater resiliency to climatic fluctuations, paradoxically, they may be more vulnerable as well. This is because of their greater dependency on groundwater resources to maintain the habitat, and their greater vulnerability to flooding.

### 3.2. Effects on Flowering, Fecundity and Survival

Although wetland flowering populations are larger than those in prairies, the greater positive response to burning in both habitats and the co-variance with climatic variation support our hypothesis that fire and climate are critical factors that drive flowering in both habitats, and that climate regulates this process through variable rates of precipitation. Fire effects on inflorescence size and fecundity were complex and not easily interpreted. Larger inflorescences in burned wetlands may relate to greater moisture availability, as suggested by a significant covariance with PDSI. However, the greater proportion of capsules formed in burned prairie and in unburned wetland may reflect other factors driving capsule formation, such as pollinator availability. Nevertheless, climate also co-varied with capsule production, which supports the finding of Wallace [43], that drought reduces fruit production.

Our analysis suggests that, as with flowering and fecundity, survival is significantly affected by interactive effects of habitat, fire, and climate. To our knowledge, almost no data have been published on this process in orchids. The comparatively higher survival rate in wetland than in prairie habitat may have resulted from reduced water stress due to greater and more stable soil moisture resulting from greater access to the water table. Likewise, the lower survival in burned prairie may have resulted from greater water stress imposed by increased soil moisture loss at lower PDSI values. This is further evidenced by the increase in survival corresponding to increasing PDSI values. The process also appears to be operating in wetlands but is probably moderated by the greater availability of soil moisture. Because we lack direct measurements of available soil moisture and direct causes of mortality such as physiological stress, these effects must be considered hypothetical. More research is needed to understand how such processes may operate in *P. leucophaea* and its associated mycorrhizal fungi.

### 3.3. Crossing Effects on Seed Germination

This study supports previous findings that *P. leucophaea* has a facultative outcrossing breeding system. This system allows for the production of viable seed from self-pollination, but higher germination percentages resulting from crosses among individuals suggests that self-pollination results in inbreeding depression [36,43]. Even higher germination values resulting from crossing between populations may result from heterosis and would require an examination of later generations to assess whether outbreeding depression eventually results from longer-distance crosses (e.g., [57,58]).

### 3.4. Effects of These Factors on Population Demography

Population viability analysis allows the integration of multiple effects on survival, flowering, and fecundity and their subsequent effect on population growth and persistence [25]. Our demographic modeling indicates that the overall positive effect of prescribed burns on flowering, fecundity, and survival, as discussed above, results in higher population growth rates and increasing population persistence with increasing fire frequency. The effect of changes on λ indicated by the demographic models, suggest that the positive effects of burning on population growth rates are apparently a result of an increase in plant growth and fecundity. Other researchers have reported that burning increases population growth rates in other prairie forbs [24,59,60]. The threshold fire frequency of 0.3 for 100-year persistence is similar to the fire frequency needed to stabilize species richness in prairie [52], which suggests that burning has similar positive effects for many prairie plants. Similarly, simulation models indicated that a fire frequency of ca. 0.2 resulted in the highest persistence probability for *Chamaecrista keyensis* (Pennell) Britton & Rose in pine rocklands in Florida [61].

Our analysis indicates a demographic switch between habitat types, in which wetland populations depend more upon survivorship than fecundity, and upland populations depend more on fecundity than on survivorship especially in response to fire. Despite the greater effect of changes in plant growth on λ for prairies compared to wetlands, demographic modeling indicates that population growth rate (λ) and persistence is greater in wetlands. This is most likely the consequence of greater survival and not fecundity since the effect of changes in fecundity are very similar in wetlands and prairies. Although juvenile stages obtain some of their carbon from soil fungi via mycotrophy, the development of leaves (photosynthesis) at a critical transition period in the orchid’s life would be expected to improve survival. The removal of detritus through burning prior to the emergence of leaves would provide these juvenile stages with more access to sunlight and greater carbon gain. Survival may be a more important demographic process in wetlands because higher soil moisture availability promotes greater survivorship during drought years. Few demographics studies compare population growth rates and persistence across soil moisture gradients. Although a North American alpine member of the Rosaceae, *Ivesia lycopodioides* A. Gray var. *scandularis* (Rydb.) Ertter & Reveal showed no significant relationship between population growth rates and soil moisture, both fecundity and survival decreased with increasing soil moisture [62].

Climate and weather impact population growth rates and persistence for *P. leucophaea*. Higher population growth rates for normal PDSI results in long-term persistence which begins to decrease with a frequency of four or more drought or wet years in a decade. Reduced persistence in drought years appears to be a consequence of lower survival, especially in prairies. The lower persistence in wet years is more difficult to explain since survival is greater in wet years. The greater effects of changes in stasis on λ in wet years suggests that the lower persistence in wet years may be predominately because plants tend to become smaller in wet years. The causes of smaller plants in wet years may be related to stress from extreme soil moisture.

Climate and weather have also been found to impact population viability in other species of orchids. Although drought and flooding differed among nine populations of *Platanthera chlorantha* (Custer) Rchb. vitality structure analysis indicates that all nine populations are thriving [6]. For the orchid *Himantoglossum hircinum* (L.) Spreng. in Germany, demographic analysis indicated that population growth increased with warmer winter temperature [63]. Demographic simulations of Ghost Orchid, *Dendrophylax lindenii* (Lindl.) Benth. ex Rolfe., populations in Cuba indicate that hurricane frequencies of 0.14 and above greatly increase the chance of population extinction [64]. Simulations also indicate that extinction risk increases with increasing hurricane severity for *Lepanthes caritensis* Tremblay & Ackerman in Puerto Rico [65].

The increase in population growth rate with increasing outcrossing and long-term population persistence appears to be best explained by the higher germination percentages caused by a shift from selfing to outcrossing. Bowles et al. [57] also reported an increase in population growth rate with increasing outcrossing in *Asclepias meadii* Torr. ex A. Gray but also detected outbreeding depression resulting from crosses between populations. Outbreeding depression has not yet been assessed in *P. leucophaea* due to the difficulty in propagating seedlings from subsequent generations.

### 3.5. The Impact of Burning on Biotic Agents—Moth Pollinators and Mycorrhizal Fungi

*Platanthera leucophaea*’s long-term survival hinges on the survival of the biotic agents needed for the orchid’s sexual reproduction, primarily pollinators and mycorrhizal fungi. Little information is available on how pollinators and fungi respond to habitat manipulation and climatic variability at the local scale, and we lack data in this study. However, fire appears to have significant effects on both agents.

Fire processes can have dynamic effects on insect populations by directly impacting different life stages present during burns [66]. However, soil surface heat generated by grassland fire dissipates almost immediately, and subsurface temperatures remain relatively cool. Although most pollinators appear to avoid fire by pupating underground, *Xylophane tersa* may pupate above ground level in leaf litter [67] and could be affected by hot surface fire. Most of the documented pollinators prefer woody plants (especially Vitaceae) as food sources which would be negatively impacted by fire; *Lintneria eremitius* prefers herbaceous plants in the Lamiaceae which occur in tallgrass prairie, while *Manduca sexta* prefers Solanaceae garden and crop plants [67]. Thus, as with other insects, unburned refugia may be important for maintaining the reproductive habitat and host plants for most of these species [68,69,70]. Due to their long-distance flights, these hawk moths would be able to access *P. leucophaea* populations that lack their larval food plants [18,71,72,73]. Information on native plants (other than *P. leucophaea*) used as nectar resources by hawk moths is lacking, and it is thus unknown whether they use other prairie plants as nectar sources. As the frequency of fire increases, plant diversity and flowering increases in prairie habitat [52,74] which can, in turn, benefit these pollinators. However, other factors beyond the control of managers may affect long-term hawk moth population persistence, including climate change, pesticides, and larval decline from parasitoids [75].

There appear to be minimal direct effects of fire on mycorrhizal fungi in soil of photosynthetic orchids as they occur below the soil surface. These fungi persist as free-living saprophytes, apparently on decomposing organic matter. However, they may be sensitive to indirect fire effects on vegetation and soil nutrients. For example, some of these fungi (e.g., *Ceratobasidium*, *Tulasnella*) exploit different nutrient sources and use ammonium as a source of inorganic N [74]. Burning affects soil nutrient cycling, including N pulses following fire [73] and may play a key role in determining mycorrhizal community composition. Moreover, Jasinge et al. [74] noted a shift in orchid mycorrhizal fungi from *Tulasnella calospora* (Bourdier) Juel to *Ceratobasidium* sp. following burning. The association of *Ceratobasidium* fungi with *P. leucophaea* in prairie habitats may correspond to the fire dependency of this habitat, how fire cycles soil nutrients, and subsequent effects on both *Ceratobasidium* and *P. leucophaea* [45]. Likewise, the closely related species, *P. praeclara*, also associates primarily with *Ceratobasidium* fungi in fire-dependent prairie habitat [75]. More work is needed to assess fungal relations of *P. leucophaea* in less fire-dependent habitats in the eastern part of its range.

## 4. Materials and Methods

We accessed data from 42 populations occurring across a moisture gradient ranging from dry-mesic (well drained) to wetland fen and sedge meadow. For statistical analysis (see below), we partitioned habitats into upland prairie (dry-mesic and mesic) and wetland (wet-mesic and wet prairie, fen, and sedge meadow). Prairie habitats included 18 populations and wetland habitats included 24 populations. All but four of these populations occurred within 50 km of Chicago, IL. One population occurred 160 km south, the second 75 km southwest, the third 120 km west, and the fourth 180 km west of Chicago. Seven of the study populations were the result of seed sown by hand between 1993–2004. One of these restored populations occurred in a site with a former record for this species; all others were novel populations. Six additional populations were excluded from the study because of their small size (<1 flowering plant average census count), infrequent appearances, and/or apparent extirpation.

Annual monitoring data were collected by volunteers initially coordinated by The Nature Conservancy from 1991–1997, then by the U.S. Fish and Wildlife Service between 1998–2020 [76]. These data were supplemented by flowering census data collected between 1980–1997 (e.g., [12]). In addition to an annual census of the number of flowering plants, demographic data were collected from >5000 permanently marked plants among 36 orchid populations between 1998–2011. These data included the fates of flowering and non-flowering plants over time, plant inflorescence size (number of flowers), and the number of capsules produced. Plants were recorded as occurring in burned or unburned habitat immediately prior to the current growing season. Though this gives a precise record of the presence or absence of fire immediately before plant growth, it does not reveal historical fire frequency which has significant effects on vegetation structure [39]. Climatic effects were based on precipitation and Palmer Drought Severity Index (PDSI) records. For the Chicago Region, monthly total precipitation was accessed from O’Hare International Airport, Cook Co., Illinois, through NOAA Regional Climate Center (https://xmacis.rcc-acis.org/ (accessed on 5 May 2021)). The four outlying populations were excluded from analysis using this data set. Monthly Palmer Drought Severity Index (PDSI) data for the Chicago Region were obtained from the NOAA National Climatic Data Center at http://www.ncdc.noaa.gov/temp-and-precip/drought/docs/palmer.pdf (accessed on 19 March 2021). Latitudinal and longitudinal coordinates were used to assign PDSI values to each site. The PDSI incorporates precipitation and temperature as well as previous monthly conditions [77], and for this reason may be less precise than monthly precipitation.

### 4.1. How Do Long-Term Trends in Sizes of Flowering Plant Populations Correspond to Habitat and Climatic Variation?

The mean annual prairie and wetland population trends spanning 41 years (1980–2020) were examined. Using Pearson’s R, we correlated prairie and wetland means with monthly growing season precipitation preceding flowering of the current year (April–June). Population trends were also correlated with post flowering growing season precipitation (July–September) of the previous year. We then used a factorial ANOVA in a General Linear Model (GLM) to test whether prairie vs. wetland habitats (N = 82 records) differed in population size in relation to departure of precipitation from the 41-year norm. Data that were not normally distributed were log transformed.

### 4.2. How Are Flowering and Fecundity Affected by Habitat and Fire and How Do They Interact with Climatic Variation?

We used analysis of co-variance (ANCOVA) in a GLM to assess effects of habitat and fire on flowering plant numbers between 1980–2014 (N = 249 records). Covariates in this test were May–September precipitation and April–August PDSI values. ANOVA was also used to assess the effects of habitat (prairie vs. wetland) and fire (burned vs. unburned) on the proportion of capsules produced relative to the number of flowering plants, exclusive of hand-pollinated plants between 1999–2011 (N = 646 records). The covariates in this test were growing season and summer PDSI values. Data were log-transformed as needed. Proportional data were arc-sin transformed.

### 4.3. How Is Plant Survival Affected by Habitat and Fire, and How Do These Affects Interact with Climatic Variation?

Survival was analyzed using Logistic Regression in a two-way interactive model with N = 693 records for plants that were initially tagged and were recorded as alive or dead the following year. Ten plants that reappeared after a year of dormancy were excluded from analysis. Binary categorical factors in the model were habitat and fire. Summer PDSI was used as a numeric variable. In this analysis, fire, and summer PDSI represent Year 1, while survival was between Year 1 and Year 2. We used this experimental design because summer PDSI was positively correlated with flowering performance the following year (see below). Also, the interactive stress between fire and drought severity would most likely affect survival the following year.

### 4.4. How Do Crossing Rates (Selfing, Within and Among Populations) Affect Seed Germination?

To determine the effects of crossing on seed viability and germination, we conducted self-pollination (95 crosses, 25 plants), outcrossing within populations (102 crosses, 22 plants), and outcrossing between populations (81 crosses, 19 plants) in 2000, 2002, and 2004. These replicates included 7 recipient and 8 donor Illinois populations with distances ranging from 7 km to 200 km between populations. Each plant received all three crossing treatments distributed evenly across the open flowers on the plant that retained pollinia and the flowers were marked with jewel tags indicating cross and donor plant ID. Seeds collected from mature capsules were surface disinfected, scarified by shaking in 0.5% NaOCl, and stratified for 8 weeks at 5 °C in SDW [46]. Seed suspension samples were removed from stratification with an eyedropper. Each sample contained ca. 100 seeds. To determine seed viability, the number of seeds containing round distinct embryos were counted with a dissecting microscope and expressed as a % of the total seeds. To assess germination, the seeds were inoculated with mycorrhizal fungi (= symbiotic germination) using standard protocols, e.g., [78]. This procedure consisted of adding seeds onto the surface of filter paper strips on an oat-based medium in sterile 9 cm diam. petri dishes that were then inoculated with a fungus (*Ceratobasidium* sp.), with 6–9 replicates per treatment and 30–160 seeds per dish. Germination was expressed as stages of seedling development for orchids following [79] and illustrated in Figure 4 in [80]. Percent Stage 1 development was expressed as the number of seeds that had reached stage 1/total No. of plated seeds. Percent Stage 2 development was the number of seeds reaching stage 2/total No. of viable seeds. Percent Stage 3 development was the number of seeds reaching stage 3/total No. of viable seeds. A two-way ANOVA using a GLM was used to test whether development to stages 1–3 differed among stages, with year and cross as factors. Percent germination was the dependent variable. Percentages were ARCSIN transformed for analysis.

### 4.5. What Are the Effects of These Factors on Population Demography?

To evaluate the effects of habitat type, burning, drought, and crossing on population growth rates and viability, demographic data from 1998 to 2011 were developed into transition matrices [81] and analyzed using PopTools 3.1.1 [82]. The plants were categorized into six stages/size classes. A leaf area index (LAI) was calculated for each plant by multiplying the number of leaves by longest leaf length. The plants were categorized as juvenile (first year of appearance and LAI ≤ 10), small vegetative (>10 and ≤30 LAI), medium vegetative (>30 and ≤100 LAI), large vegetative (>100 LAI), flowering, and dormant. The three vegetative size classes were based on differences in survival and flowering for the classes: vegetative plants with LAI < 30 had significantly lower survival compared to larger plants (R_N_^2^ = 0.004, df = 4, *p* < 0.001); and vegetative plants with LAI > 100 had significantly higher flowering probability (F = 17.867, df = 3, 803, *p* < 0.001).

Transition matrices were created using multinomial logistic regression in SPSS version 12 to determine the probability of transition from a stage in year *t* to a stage in year *t* + 1. Fecundity was estimated by multiplying the mean number of capsules per flowering plant (calculated spanning all years by burn treatment, habitat type, and drought effects) by the number of seeds per capsule (≈4000; L. Zettler, unpublished data), by the proportion of viable seeds, and by the proportion of viable seeds that reach protocorm Stage 5 [leaf elongation]. All matrices used the outcrossed within proportion of viable seeds and proportion reaching Stage 5 except the outcrossed between and selfed matrices which used the proportion of viable seeds and proportion reaching Stage 5 for outcrossed between and selfed crosses, respectively.

Finite population growth rates (λ) and the 95% confidence interval around λ were calculated in R Version 1.3.1093 [83] using the Delta method [84] (see [57] for further details). Persistence and extinction probability were determined by simulation using RAMAS Metapop version 4.0 [85]. All simulation parameters included 1000 replications, for 100 1-year time-steps, extinction threshold of 0, demographic stochasticity, lognormal environmental stochasticity, experimental density dependence, and uncorrelated vital rates. Initial abundances were equal to the stable stage distribution of the pooled unburn transition matrix (367, 66, 22, 1, 21, 23). The mean age of parents of cohort juveniles was calculated using PopTools 3.1.1 [82].

To examine the effects of drought on population growth rates and viability, growing season Palmer Drought Severity Index was used to categorize pairs of transition years into drought (PDSI < −1), normal (PDSI between −1 and 1) and wet (PDSI > 1). The effect of drought or wet year frequency was modeled using normal year transitions for the transition matrix and standard deviation matrix. Drought and wet year frequency effects were modeled using stage specific multipliers calculated by dividing either drought year by normal or wet year by normal for each transition frequency [85]. Thus, transitions for which normal was lower had stage specific multipliers above 1. Drought and wet year frequency were modeled in RAMAS using a frequency ranging from 0.0–1.0 in 0.1 increments.

For comparison of burn effects, the model used the transition matrix and standard deviation matrix of the unburned transitions. Prescribed burns were modeled using stage specific multipliers calculated by dividing burn by unburn for each transition frequency [85]. Prescribed burn was modeled in RAMAS using a burn frequency ranging from 0.0–1.0 in 0.1 increments for a total of eleven separate models.

To evaluate the effect that changes to groups of stage transitions have on λ [86], we grouped elasticities of stage transitions into the lower left region of the transition matrix representing the combined effects of changes to growth on λ, the diagonal and upper right region representing the combined effects of changes to stasis and retrogression on λ, and the fecundity (top) region representing the combined effects of changes to recruitment on λ.

## 5. Conclusions

More than 50% of all orchids that are native to the U.S. and Canada are currently listed as threatened or endangered (https://northamericanorchidcenter.org/, accessed on 5 May 2021) and among these is *Platanthera leucophaea*, an appealing and well-known species with a range that overlaps both countries. Until now, research on *Platanthera leucophaea* has lacked long-term monitoring data and modeling needed to link ecological drivers with demography. This current study provides such information for conservation of *P. leucophaea* in the prairie region comprising the western part of its distribution. We demonstrate that both habitat (prescribed burning) and population (outcrossing) management can improve eastern prairie fringed orchid population viability, and that habitat conditions and climatic variation interact with these effects. Our data also demonstrate a demographic switch between habitat types, driven by interactions between habitat and climatic variation. Wetland populations depend more upon survivorship than fecundity, while, conversely, upland prairie populations depend more on fecundity than on survivorship. This is illustrated in long-term census data, where wetlands have lower proportional fruit production, and respond more rapidly than upland prairies following drought extremes due to greater survival of vegetative plants. Moreover, the long-term persistence of this species in fragmented habitats may be greater in wetlands, and wetlands may supplement post drought recovery in upland sites. Future climate change may reinforce this difference if it impacts upland sites to a greater degree than wetlands. However, parallel studies are still needed in the other habitats such as wetlands, especially in the eastern part of the range of the species, to provide a more complete picture. More information is also needed to document and safeguard the mycorrhizal fungi from these other sites, and techniques are still needed that improve *P. leucophaea*’s artificial propagation from seed. Clearly, the conservation of this ephemeral species is an ongoing process that requires long-term monitoring. Underpinning our analysis was the coordinated action of a network of volunteers who were mobilized annually to collect monitoring data. These volunteers epitomize a successful partnership that made it possible for us to analyze the high quality, long-term monitoring data that culminated in this paper.

## Figures and Tables

**Table 1:** *Platanthera leucophaea* population growth rates, persistence and extinction duration for burn treatment, drought effects, crossing effects and habitat type. Values with different letters within a group are significantly different at *p* = 0.05.

		Population Growth Rate (λ)	95% CL	Mean Persistence (years)	se	Mean Extinction Duration (years)	se
Pooled		1.10529	0.25694	100	0	0	0
Burn effects	unburn	1.10732 ^a^	0.05423	100 ^a^	0	0 ^a^	0
	burn	1.63042 ^b^	0.03619	100 ^a^	0	0 ^a^	0
Drought Effects	drought	0.62124 ^a^	0.12823	13.0 ^c^	0.30	87.0 ^c^	0.39
	normal	1.10996 ^b^	0.11775	100 ^a^	0	0 ^a^	0
	wet	0.71422 ^a^	0.22808	15.9 ^b^	0.56	84.1 ^b^	0.56
Crossing effect	outcross within	1.10529 ^b^	0.25694	100 ^a^	0	0 ^a^	0
	outcross between	1.73496 ^a^	0.44092	100 ^a^	0	0 ^a^	0
	self	0.88622 ^c^	0.19454	27.1 ^b^	0.92	72.9 ^b^	0.92
Habitat type	wetland	1.29147 ^a^	0.16234	75.0 ^a^	3.21	21.8 ^a^	3.10
	prairie	0.83853 ^b^	0.27830	9.4 ^b^	0.44	90.6 ^b^	0.44

## Data Availability

Publicly available datasets were analyzed in this study.
